# Outcomes and Blood Product Use in 89 Surgically Managed and 79 Medically Managed Cases of Acute Spontaneous Hemoperitoneum in the Dog

**DOI:** 10.3389/fvets.2021.736329

**Published:** 2021-10-08

**Authors:** Leah Veronica Wright, Michelle Renwick, Rachel W. Y. Soh, Nicole R. Fan, Anna J. Tebb, Yenny H. Indrawirawan

**Affiliations:** ^1^Emergency and Critical Care Department, Veterinary Referral Hospital, Melbourne, VIC, Australia; ^2^Faculty of Veterinary and Agricultural Sciences, The University of Melbourne, Parkville, VIC, Australia; ^3^Emergency and Critical Care Department, The University of Sydney, Darlington, NSW, Australia; ^4^Emergency and Critical Care Department, Sydney Veterinary Emergency and Specialists, Sydney, NSW, Australia; ^5^Small Animal Medicine Department, Western Australian Veterinary Emergency and Specialty, Perth, WA, Australia

**Keywords:** hemoperitoneum, canine, transfusion, surgery, hemangiosarcoma (HSA), stabilization

## Abstract

**Objective:** To describe a population of dogs with acute spontaneous hemoperitoneum (ASH) that were treated with either surgical or medical management in the first 12 h after presentation, and to perform a preliminary investigation into whether there were differences in achieving cardiovascular stabilization or patterns of red blood cell (RBC) transfusion between patients treated with early (<12 h) surgery vs.medical management.

**Design:** A retrospective multicenter preliminary study performed on 168 dogs presenting with ASH between January 2015 and May 2019. Patients were excluded if they were euthanized or discharged from hospital within the first 12 h, or if clinical records were incomplete. All patients received appropriate medical stabilization efforts. Statistical analysis was performed comparing patients that underwent early (<12 h) surgery and those that did not.

**Results:** Eighty-nine patients were in the early surgical group and 79 patients in the medical group. A significantly higher proportion of medical cases were euthanized (*p* < 0.001). A significantly higher proportion of early surgical cases were discharged from hospital (*p* = 0.005). There was no statistically significant difference between groups in achieving cardiovascular stabilization (OR 1.07 *p* = 0.82). A higher proportion of patients with body-weight over 20 kg achieved stabilization within 12 h than those with body-weight of 20 kg or less (62.7 vs. 41.4%, *p* < 0.01). A higher proportion of patients with splenic conditions achieved stabilization than patients with non-splenic conditions (56.5 vs. 28.6%, *p* = 0.05). The odds of receiving an RBC transfusion were higher in the early surgical group than the medical group [OR 3.81 (*p* < 0.001)].

**Conclusions:** This preliminary study did not identify a significant difference in the ability to achieve cardiovascular stabilization in the first 12 h in dogs with ASH that underwent early surgical intervention vs. those managed medically. Patients in the early surgical group were more likely to receive a RBC transfusion than those in the medical group. At this time the decision on whether to pursue medical or early surgical management should be made on a case by case basis.

## Introduction

Canine Acute Spontaneous Hemoperitoneum (ASH) is a common, potentially life-threatening emergency (1), and owners of these patients are often faced with the urgent need to make difficult emotional and financial decisions. There is no currently recognized consensus among veterinary criticalists and surgeons as to whether immediate surgical intervention is necessary to achieve short-term stabilization in these patients, and because of this, the decision between medical and surgical treatment is commonly based on clinician experience and hospital protocols.

Commonly reported causes of canine acute spontaneous hemoperitoneum (ASH) include intra-abdominal neoplasia, hematomas, liver lobe torsion, splenic torsion, gastric dilation and volvulus and coagulopathies (2–4). Recent case reports and retrospective studies have also identified ASH associated with anaphylaxis without concurrent coagulopathy (5–7). ASH is a rare condition in humans, but most commonly arises from hepatic, splenic, vascular, or gynecological pathology (8). Numerous studies have found malignant neoplasia, frequently originating from the spleen, to be the most common cause of spontaneous hemoperitoneum in dogs (3, 4, 9). Splenic hemangiosarcoma causes the greatest proportion of ASH cases (10, 11); however, a recent study by Fleming et al. found dogs under 20 kg to be more likely to have hemoperitoneum of non-splenic origin, and less likely to have an etiological diagnosis of hemangiosarcoma than dogs ≥20 kg (4).

ASH requires prompt and appropriate intervention, and some authors consider ASH due to ruptured masses, hematomas and organ torsions to be indications for early surgical intervention (12, 13). Surgical exploration is explicitly contraindicated in cases of ASH due to coagulopathy and known anaphylaxis (5–7). Although many owners report an acceptable quality of life following surgical intervention for hemoperitoneum due to neoplastic abdominal masses (14), emergency surgery may be associated with increased risk of perioperative mortality (15), and common etiologies such as hemangiosarcoma can carry poor long term prognoses (3, 9). These reasons, along with the high financial cost of emergency surgery have prompted some clinicians to consider whether medical stabilization alone may be appropriate short-term management for cases of ASH. Similarly, the costs associated with blood product administration, and in some cases difficulty in accessing blood products, can be determinants in whether owners pursue treatment or euthanasia for cases of ASH. As such, the purpose of this preliminary retrospective study is to describe a population of dogs with ASH that were treated with either surgical or medical management, and to perform a preliminary investigation into whether there were differences in achieving cardiovascular stabilization or patterns of blood product administration between patients treated with early (<12 h) surgery vs. initial medical management. Our findings will thereby be of benefit to veterinarians in their clinical decision making in management of cases of canine ASH.

## Methods

The medical databases of five Australian 24-h emergency and specialty hospitals were searched for dogs presenting with ASH between January 2015 and May 2019. Specific search parameters differed between practices depending on the individual practice management software utilized. Inclusion criteria were (1) the presence of free peritoneal fluid identified on either abdominal ultrasound or CT scan, (2) non-clotting, grossly hemorrhagic fluid retrieved from peritoneal cavity by either abdominocentesis or exploratory laparotomy, (3) no known or suspected history of major trauma, and (4) no evidence of a primary coagulopathy. Measurement of the packed cell volume (PCV) of the abdominal fluid was not necessary for inclusion, however any patient with a fluid analysis inconsistent with hemorrhage was excluded. Primary coagulopathy was defined by either prothrombin time and/or activated partial thromboplastin time >50% higher than the upper limit of the reference interval on one measurement after admission, or platelet count <30 × 10^9^/L on one measurement after admission. Exclusion criteria were either elective euthanasia or discharge from hospital within the first 12-h after admission, as cardiovascular stability could not be appropriately assessed in these patients. Patients that had been admitted with the intention to treat, but subsequently died within the first 12-h period were included in the study.

Patient signalment was recorded including age, sex, breed, neuter status, and body weight. Histopathological diagnosis, including site and etiology was recorded for all patients that underwent surgery at any time during hospitalization. Frequency of patient monitoring was determined by individual ICU protocols and the patient's clinical condition. Clinical records were reviewed to determine whether the patient was considered to be cardiovascularly stable at (1) presentation, and (2) any point during the first 12-h after presentation to hospital. A patient was considered cardiovascularly stable if they had (1) heart rate of ≤ 120 beats per minute (16), (2) systolic blood pressure of ≥90 mm Hg or mean blood pressure of ≥60 mm Hg (17), and (3) blood lactate of ≤ 3.0 mmol/L (18). Lactate could be measured on either a hand-held lactate meter or blood-gas analyzer, depending on the clinic's protocol and facilities. Patients who did not have both blood pressure and lactate reported within the first 12 h were considered stable if two out of three parameters were measured and were within the acceptable limits. If a patient had any of these criteria outside the acceptable range at all recorded time points, or if they died during the first 12 h, they were considered “unstable.” Patients with incomplete clinical records were excluded from the study.

The volume of whole blood or packed red blood cells (pRBC) administered to each patient during the entire duration of hospitalization was determined from the medical records and recorded as mL/kg bodyweight. If a whole blood transfusion was administered, a conversion factor of 0.75 was applied to the volume administered in order to account for the higher PCV in pRBC [~60% (19)] compared to whole blood [~45%, normal range 37–55% (20)]. This conversion was used to determine the equivalent volume of packed red blood cells (EpRBC). PCV was recorded at presentation.

Patients that underwent definitive surgical intervention e.g., exploratory laparotomy within the first 12-h period were included in the “early surgical” group. Patients who received only medical management within the first 12-h period were included in the “medical” group. These groups were used as the basis for descriptive analysis of the entire patient population. “Medical” patients that went on to have surgery after the first 12 h of hospitalization were included in the “late surgery” sub-group.

The “early surgical” group was categorized based on the reason for early surgery—either planned, patient decompensation, or reason unable to be determined. The planned early surgery subgroup included patients taken to surgery based on hospital protocol or due to clinician or owner preference. The decompensated subgroup included patients that continued to show clinical deterioration despite initial medical management. In some cases, the reason for surgery was unable to be determined due to insufficient information in the clinical history.

Statistical analyses were carried out in R version 4.0.0 (21); packages stats, dplyr, epiR, and descr. The level of significance was set at 0.05 throughout. Demographics and clinical variables were reported. Continuous variables were assessed for normality visually and medians were calculated for skewed variables.

The odds of stabilization and receiving a transfusion were compared between the “early surgical” group and the “medical” group by multivariable logistic regression. The two groups were tested for difference with respect to variables. Variables with *p*-values of 0.3 or less were included for consideration in the multivariable model. Model building was conducted by backwards elimination until all variables remaining were significant at the 0.05 significance level. Adjusted odds ratios were reported.

Further comparisons between proportions of outcomes and etiologies were compared using Chi-squared test or Fisher's exact test as appropriate and comparisons between continuous variables using Mann-Whitney *U*-test.

## Results

One hundred and ninety-one cases of ASH presenting between 2015 and 2019 were eligible for inclusion in the study. Twenty-three cases were excluded due to incomplete clinical records, leaving 168 cases in the final analysis. There were no significant differences identified between the population of cases excluded and those remaining in the study, and therefore no bias appears to have be introduced by removing these patients from the analysis.

Eighty-nine patients were in the “early surgical” group and 79 were in the “medical” group. Eighty-five cases originated from clinic A, seven from clinic B, 13 from clinic C, 33 from clinic D and 30 from clinic E. Fifteen cases were considered stable on presentation—eight in the “early surgical” group and seven in the “medical” group.

Patient demographics are shown in Table 1. The most commonly represented breeds were the German Shepherd (*n* = 20), Labrador retriever (*n* = 19), and Staffordshire Bull Terrier (*n* = 14). The median age was 10.66 years (range: 15 weeks−16.0 years), and median weight was 25.65 kg (range: 4.70–55.0 kg). The majority of dogs were neutered (89.9%).

**Table 1:** Patient demographics.

**Variable**	**All participants**	**Early surgical**	**Medical**
	**(*n =* 168)**	**(*n =* 89)**	**(*n =* 79)**
Age [median (range)] (years)	10.66 (0.29–16.0)	11.0 (0.29–15.0)	10.0 (6.0–16.0)
Weight [median (range)] (kg)	25.65 (4.7–55.0)	29.0 (4.8–51.0)	22.1 (4.7–55.0)
**Breed group:**
Group 1: Toys	11 (6.5)	3 (3.4)	8 (10.1)
Group 2: Terriers	18 (10.7)	12 (13.5)	6 (7.6)
Group 3: Gundogs	34 (20.2)	22 (24.7)	12 (15.2)
Group 4: Hounds	14 (8.3)	9 (10.1)	5 (6.3)
Group 5: Working	38 (22.6)	17 (19.1)	21 (26.6)
Group 6: Utility	12 (7.1)	4 (4.5)	8 (10.1)
Group 7: Non-sporting	9 (5.4)	5 (5.6)	4 (5.1)
Crossbreeds	32 (19.0)	17 (19.1)	15 (19.0)
**Sex**
Male [*n* (%)]	86 (51.2)	51 (57.3)	35 (44.3)
Female [*n* (%)]	82 (48.8)	38 (42.7)	44 (55.7)
**Neuter status**
Neutered [*n* (%)]	151 (89.9)	73 (82.0)	78 (98.7)
Entire [*n* (%)]	17 (10.1)	16 (18.0)	1 (1.3)

*Breed groups according to Australia National Kennel Council (available from: ankc.org.au, accessed 05/03/2020)*.

Patients in the “early surgical” group had significantly higher body weight (median 29.0 kg, range 4.8–51.0 kg) than patients in the “medical” group (median 22.1 kg, range 4.7–55) (*p* = 0.001), and a smaller proportion were neutered (82 vs. 98.7%) (*p* < 0.001). There were no significant differences in sex, breed or age between groups. There was also no difference in presenting PCV between groups; median (range) in early surgical cases was 31 (13–58%) and in medical cases was 30 (16–52%); *p* = 0.42 (Table 2).

**Table 2:** Presenting PCV between “early surgical” (*n* = 89) and “medical” (*n* = 79) groups.

**Presenting PCV**	**Early surgical**	**Medical**
Median (range)	31 (13–58%)	30 (16–52%)
0–25% [*n* (%)]	26 (29.2)	26 (32.9)
>25–30% [*n* (%)]	16 (18.0)	15 (19.0)
>30–40% [*n* (%)]	29 (32.6)	27 (34.2)
>40% [*n* (%)]	18 (20.2)	11 (13.9)

**Figure 1:**
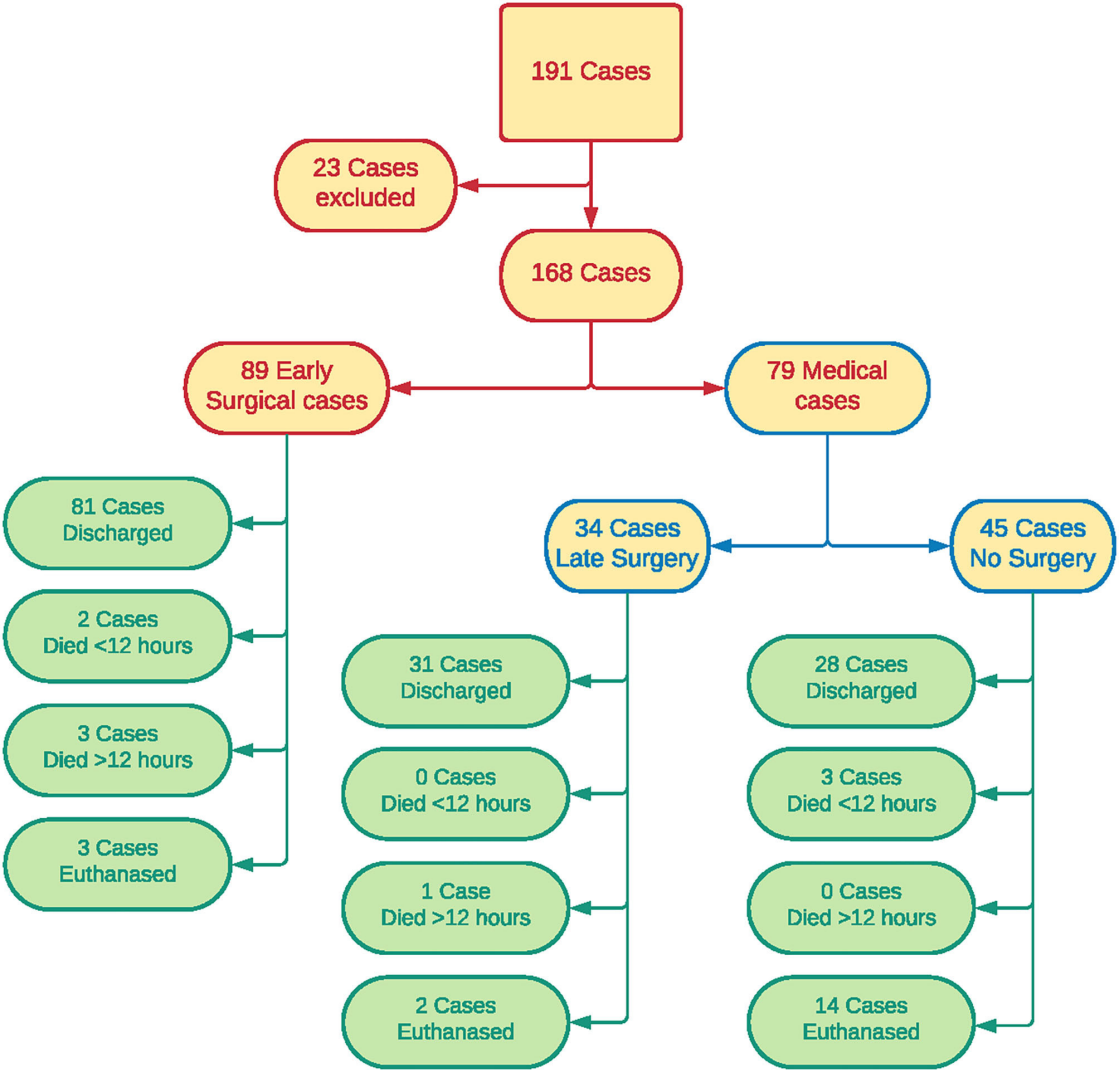
Patient outcomes.

Patient outcomes are shown in Figure 1. Of patients in the “early surgical” group, 60 had surgery due to protocol, 20 due to clinical decompensation, and the reason could not be determined in nine cases. Of the 79 patients in the “medical” group, 34 went on to have surgery after the initial 12-h period (late surgery sub-group). All 34 of these dogs underwent planned surgery after staging, and none were determined to be decompensating.

Forty-five patients did not have surgery at any time during hospitalization. Sixteen were transferred to other veterinary clinics for ongoing management which may have included surgery, 12 were discharged for palliative care, 14 were euthanized and three died in the first 12 h of medical management. Of the 28 “medical” patients that were transferred to other clinics or discharged for palliative care, 21 were considered stable at the time of discharge, six were unstable, and there was insufficient detail in the clinical record to assess cardiovascular stability at discharge in one patient.

One hundred and forty patients were discharged from hospital, including 81 “early surgical” patients and 59 “medical” patients. A significantly higher proportion of “early surgical” cases were discharged from hospital [χ^2^(1, *N* = 168) = 8.0332; *p* = 0.005]. In total, 19 patients were euthanized; 16 in the “medical” group and three in the “early surgical” group. A significantly higher proportion of “medical” cases were euthanized [χ^2^(1, *N* = 168) = 11.892; *p* < 0.001]. Five dogs were euthanized after surgery, nine were euthanized due to poor prognosis after staging, and five were euthanized due to owners declining further investigation or treatment beyond initial stabilization.

Nine patients died in hospital, including five out of 89 “early surgical” patients and four out of 79 “medical” patients. Five of these patients (two early surgical, three medical) died within the first 12 h, and none of these had been considered stable at any time point. Four patients (three early surgical, one medical) died after the first 12 h of hospitalization. Two of these four patients had been considered stable during the first 12 h (one early surgical, one medical). There was no significant difference in the proportion of patients that died <12 h (*p* = 0.667) or >12 h (*p* = 0.623) between “early surgical” and “medical” patients.

In dogs that underwent surgery, the spleen was the most common site of hemorrhage (107/123 dogs), and splenic hemangiosarcoma was the most common histopathological diagnosis (58/123 dogs) (Table 3). Hemorrhage originated from the liver in 11 dogs, the adrenal gland in two dogs and a retroperitoneal mass in one dog. There was no significant difference in site or etiology of hemorrhage between patients that received either early or late surgery.

**Table 3:** Etiology of cases which had early (<12 h) (*n* = 89) vs. late surgery (*n* = 34).

**Anatomical location**	**Pathology**	**Total frequency**	**Early surgery frequency**	**Late surgery frequency**
		**[*n* (%)]**	**[*n* (%)]**	**[*n* (%)]**
Adrenal gland		2 (1.6)	1 (1.1)	1 (2.9)
	Unspecified	2 (1.6)	1 (1.1)	1 (2.9)
Liver		11 (8.9)	9 (10.1)	2 (5.9)
	Hemangiosarcoma	2 (1.6)	1 (1.1)	1 (2.9)
	Benign hepatic mass	4 (3.3)	3 (3.4)	1 (2.9)
	Other malignant hepatic mass	1 (0.8)	1 (1.1)	0 (0.0)
	Torsion	2 (1.6)	2 (2.2)	0 (0.0)
	Unspecified	2 (1.6)	2 (2.2)	0 (0.0)
Retroperitoneal		1 (0.8)	1 (1.1)	0 (0.0)
	Hemangiosarcoma	1 (0.8)	1 (1.1)	0 (0.0)
Spleen		107 (87.0)	76 (85.4)	31 (91.2)
	Hemangiosarcoma	58 (47.2)	41 (46.1)	17 (50.0)
	Benign splenic mass	38 (30.9)	28 (31.5)	10 (29.4)
	Other malignant splenic mass	6 (4.9)	3 (3.4)	3 (8.8)
	Unspecified	5 (4.1)	4 (4.5)	1 (2.9)
None found		1 (0.8)	1 (1.1)	0 (0.0)
Unspecified		1 (0.8)	1 (1.1)	0 (0.0)
**Total**		123 (100.0)	89 (100.0)	34 (100.0)

Splenic hemangiosarcoma occurred significantly less frequently in dogs ≤ 20 kg compared to dogs >20 kg, χ^2^(1, *N* = 122) = 5.184 (*p* = 0.023). There was no significant difference in the prevalence of hepatic hemorrhage or hemorrhage from other sites between body weight groups. In one case the site of hemorrhage was not recorded in the clinical history, and in another patient no source of hemorrhage was found at surgery.

There was no statistically significant difference between the “early surgical” group and the “medical” group in achieving cardiovascular stabilization [Odds ratio 1.02 (95% CI 0.53, 1.97) *p* = 0.94] (Table 4).

**Table 4:** Contingency table for stabilization between “early surgical” (*n* = 89) and “medical” (*n* = 79).

**Surgery group**	**Total**	**Early surgical**	**Medical**
Stabilized [*n* (%)]	93 (55.4)	50 (56.2)	43 (54.4)
Not stabilized [*n* (%)]	75 (44.6)	39 (43.8)	36 (45.6)
Total [*n* (%)]	168 (100.0)	89 (100.0)	79 (100.0)

A higher proportion of patients with bodyweight over 20 kg achieved stabilization within 12 h than those with bodyweight of 20 kg or less (62.7 vs. 41.4%) [χ^2^(1, *N* = 168) = 7.003; *p* < 0.01]. A higher proportion of patients with splenic conditions achieved stabilization compared to patients with non-splenic conditions (56.5 vs. 28.6) [χ^2^(1, *N* = 122) = 3.878; *p* = 0.05].

The odds of receiving an RBC transfusion were higher in the “early surgical” group than the “medical” group [Odds Ratio 3.81 (1.92, 7.58) (*p* < 0.001)]. For the 79 patients that received an RBC transfusion, the median EpRBC was 14.5 mL/kg (range 6.0–76.3 mL/kg) (Table 5). When considering only those patients that received a transfusion, there was no significant difference in EpRBC between treatment groups (*p* = 0.835).

**Table 5:** Transfusion requirements between “early surgical” (*n* = 89) and “medical” (*n* = 79) groups.

**Variable**	**Early surgical**	**Medical**		
		**Total**	**Late surgery**	**No surgery**
Transfused [*n* (%)]	53 (59.6)	26 (32.9)	12 (35.3)	14 (31.1)
Not transfused [*n* (%)]	36 (40.4)	53 (67.1)	22 (64.7)	31 (68.9)
EpRBC (mL/kg) (Patients receiving a transfusion only) (median (range))	15.3 (6.0–76.3)	13.9 (6.4–36.8)	13.4 (6.4–23.4)	16.8 (7.5–36.8)

## Discussion

Our preliminary study found no significant difference in the odds of achieving cardiovascular stabilization in dogs with ASH, whether they were managed with surgical or medical management within the first 12 h of presentation. Dogs over 20 kg were more likely to achieve stabilization than dogs ≤ 20 kg, and dogs with hemorrhage originating from splenic lesions more likely to achieve stabilization than those with non-splenic sources of hemorrhage.

There was no significant difference in in-hospital mortality between the two groups, and dogs receiving early surgical intervention were more likely to receive a blood transfusion than the medically managed group. To the authors' knowledge, this study is the one to investigate the impact of early surgical management vs. medical management on the outcomes of cardiovascular stabilization and transfusion requirements in canine ASH. Failure to detect a difference in stability or mortality may be a type two statistical error due to the small sample size.

Our findings suggest that it may be appropriate to pursue aggressive medical stabilization in patients with ASH, and that early surgical intervention could be reserved for those patients that do not stabilize with medical interventions alone. Furthermore, short term medical management can be recommended in instances where owners wish to pursue palliative care, or elect to transfer the patient for ongoing management or surgical intervention elsewhere. The potential benefits of delaying surgery pertain mostly to cases that present out of hours. Of the 79 patients in the “medical” group, 12 were discharged for palliative care and 16 were transferred to other veterinary clinics for ongoing management.

Consistent with previous studies (3, 4), our results found splenic hemangiosarcoma to be the most common cause of ASH. This is a malignant neoplasia which frequently metastasizes prior to development of ASH, and which has median post-operative survival times of only 3–12 weeks with surgery alone (22). Overall, we found malignant disease was present in 55.3% of patients that received a definitive diagnosis. For this reason, a thorough investigation for metastatic disease is recommended prior to surgical intervention for ASH whenever possible.

Plain radiography is a relatively insensitive tool for detecting early metastases (23, 24), particularly in the emergent situation where patient positioning, exposure quality and number of projections may be compromised. Therefore, additional diagnostic imaging techniques should be considered when available, in order to provide a more sensitive assessment of potential metastases. Specialist ultrasound can frequently detect both the primary cause of ASH and metastatic spread within the abdomen; however, identification of multiple abdominal lesions is not necessarily associated with a diagnosis of malignancy in dogs with ASH (25). Computed tomography (CT) is the preferred imaging modality of oncologists for staging metastatic disease (26). Furthermore, CT is the standard diagnostic tool used in the management of hemoperitoneum in humans (1) and is also the gold standard diagnostic test for detection of pulmonary metastases in dogs (24, 26). It allows excellent evaluation of abdominal organs for both primary and metastatic lesions, and is highly sensitive for detection of even subtle pulmonary lesions (26, 27).

CT may be superior to ultrasound at screening for abdominal disease in dogs >25 kg (28). Given that the median weight of dogs in the current study was 25.65 (range 4.70–55.00), CT alone, or in combination with specialist ultrasound, may increase the sensitivity of detection of both pulmonary and abdominal metastases in many ASH patients. These diagnostic imaging findings may help owners to make a more informed decision on whether or not to undertake surgical intervention in dogs with ASH. Additionally, CT may help to identify the source of hemorrhage pre-operatively through identification of a mass lesion and the sentinel clot sign (29). This may be of particular benefit for surgical planning in patients with multiple intra-abdominal mass lesions.

One dog in our cohort had no anatomical source of hemorrhage identified at surgery. Hemorrhage secondary to anaphylaxis has recently been reported in dogs (5–7), and should therefore be considered as a possible cause of ASH in this patient. Given the absence of gross disease at exploratory surgery, a more thorough diagnostic imaging investigation or serial abdominal FAST scanning may have prevented unnecessary surgical intervention in this patient.

Many clinics are limited in their advanced diagnostic imaging capabilities out-of-hours. Our findings suggest that it may be both safe and warranted to postpone definitive surgical intervention for out-of-hours presentations of ASH in order to facilitate a more thorough diagnostic investigation of the primary disease process and the presence of possible metastases. Medical stabilization may also afford owners additional time to process information regarding likely underlying etiologies and prognosis, allowing them to make a more informed decision on treatment options. This approach has the potential benefit of reducing client stress without compromising patient safety.

Out-of-hours surgery may increase the risk of anesthetic complications and peri-operative mortality. In 2018 Stefani and colleagues published a retrospective cohort study of 11,563 human anesthetic procedures which found that out-of-hours surgery (between midnight and 07:00) was significantly associated with an increased risk of early (<48 h) mortality (15). Studies on equine perioperative mortality have found a similar increased risk of death associated with out-of-hours anesthesia (30) and so it is reasonable to suspect the same is true for canine patients. The study by Stefani and colleagues also found that patients undergoing an exploratory laparotomy or an emergency surgery were similarly at an increased risk of peri-operative death (15). Uncontrolled hemorrhage and anesthesia related complications were found to be common causes of early death in these cohorts (15). We suggest that for these reasons, clinicians should consider delaying anesthesia and surgery in dogs presenting with ASH out-of-hours, as this may decrease the risk of peri-anesthetic complications and mortality.

Perioperative mortality risk is multifactorial, however Stefani et al. showed that the patient's condition and surgical complications are the most relevant contributing factors. Therefore, we propose that it is preferable to avoid general anesthesia and surgery until the patient is cardiovascularly stable whenever possible. A medical approach to ASH should only be recommended if the patient is able to be hospitalized in a 24-h facility, however. This is because ASH carries potential for acute deterioration warranting immediate reassessment of the treatment plan at any time. Rigorous patient monitoring and the ability to provide aggressive medical support and/or urgent surgical intervention may reduce the risk of short-term complications including death.

Immediate surgical intervention may be necessary in emergent patients that are deteriorating despite aggressive medical therapy. Clinical tools to identify such patients could include close patient monitoring, electrocardiography and blood pressure monitoring, serial laboratory testing such as repeat PCV/TP and lactate measurements, and serial abdominal FAST ultrasound. Serial abdominal FAST ultrasound, for example, can allow clinicians to semi-quantify hemorrhage (31) which may help guide treatment options. In our study 20 dogs underwent emergency surgery due to deterioration despite medical management. Further investigation of the specific details leading to clinicians electing emergent surgery was, unfortunately, beyond the scope of this paper.

Our results showed that early surgical intervention (<12 h) was strongly positively associated with the administration of a red blood cell transfusion (*p* < 0.001). When considering only those patients administered a transfusion; however, the EpRBC volume infused was not significantly different in the “early surgical” population compared to the “medical” population. While it is possible that a subset of patients in the “early surgical” group was inherently more critical or more difficult to stabilize than those managed medically, the presence of multiple confounding factors means that no cause-effect relationship can be inferred by this finding. In the present study, no significant difference was found in regard to cardiovascular stabilization or presenting PCV between the ‘'early surgical” and “medical” groups to account for the difference in transfusion requirement.

Due to the retrospective nature of the study, the individual attending veterinarian's clinical reasoning for administration of pRBC transfusion was not able to be determined. The cause for more frequent transfusion administration in “early surgical” patients is likely to be multifactorial, including underlying disease severity, the impact of anesthesia and surgery in an unstable patient. “Early surgical” patients also may have been preferentially resuscitated with blood products rather than crystalloids or synthetic colloids in anticipation of ongoing peri-operative hemorrhage. It is also highly likely that clinician preferences and individual clinic protocols contributed to this finding. The high proportion of “medical” patients that were either euthanased or discharged without staging or surgery suggests that owner preferences and financial limitations are also likely to have contributed to the difference in transfusion administration. Furthermore, those clinics with more ready access to blood products may have been more liberal with transfusion administration. Results show that clinic E administered significantly greater volumes of EpRBC than any other center. The majority of cases from this clinic were in the “early surgical” group which may have skewed the results for EpRBC volume infused. These findings highlight the complexity of factors affecting blood product administration, and relevance for patient outcomes.

Administration of allogenic blood products can be a lifesaving intervention in patients with acute blood loss; however, the cost of transfusion can present a major financial burden, and blood product administration has potential to cause detrimental short- and long-term effects (19). There is always a small but important risk of acute and delayed transfusion reactions (19), and administration of greater total volumes of pRBC has been associated with increased incidence of multiple organ dysfunction syndrome, sepsis, disseminated intravascular coagulation, and thromboembolic disease (32). Furthermore, transfusion related immunomodulation (TRIM) is a recently described consequence of allogenic blood product administration in man, although the exact mechanism by which this phenomenon occurs remains to be determined (33). TRIM may have as yet unknown implications in the veterinary field, and a recent study investigating long-term postoperative effects of administration of allogeneic blood products in dogs with hemangiosarcoma concluded that dogs administered allogenic blood products had a shorter disease-free interval than dogs that did not (34). For these reasons, avoidance of allogenic blood products may be of benefit in those patients that are able to be stabilized without the need for transfusions. Our findings suggest that it may be possible to reduce transfusion requirements and the associated risks by attempting medical management in some cases of ASH.

Dogs in the “early surgical” group were found to have a significantly greater body weight and were less likely to be neutered than dogs in the “medical” group. Fleming and colleagues found dogs under 20 kg were more likely to have ASH of non-splenic origin, and less likely to have hemangiosarcoma as the primary cause than dogs over 20 kg (4). In the same study, dogs with splenic causes of hemoperitoneum were more likely to undergo surgical intervention. In agreement with the findings of Fleming et al. (4), our results found splenic hemangiosarcoma to be a less frequent cause of ASH in small dogs ( ≤ 20 kg) compared to large dogs (>20 kg). Factors such as body weight, suspected anatomical location and likely etiology may have influenced decisions relating to early surgical intervention in some cases.

The difference in neuter status between groups is unlikely to have affected the results. The majority (89.9%) of patients in our study were neutered. This is consistent with the overall rates of neutering among dogs in the geographic region where the study was performed (35), and is therefore unlikely to represent a true risk factor for ASH.

We found that a significantly higher proportion of “medical” cases were euthanized, and a significantly higher proportion of “early surgical” cases were discharged from hospital. This may be an inaccurate representation of overall outcomes in patients with ASH however, as “early surgical” patients that were euthanized intraoperatively during the first 12 h of hospitalization were excluded from the study. In the “medical” group, nine patients were euthanased due to poor prognosis after staging. It is possible that some of the “early surgical” patents would have been euthanased for the same reason had more thorough diagnostic imaging been pursued preoperatively. Similarly, some of the excluded cases may have been euthanased within the first 12 h due to evidence of gross metastases identified at surgery. A further five “medical” patients were euthanased because the owners declined further staging or surgical intervention. The decisions of these owners may have been influenced by the additional time allowed to process information regarding likely etiology and prognosis. While a greater proportion of “early surgical” patients were discharged from hospital, many of these patients were diagnosed with a malignant cause of hemoperitoneum and long term follow up was beyond the scope of this paper.

There are several limitations to this study, mainly associated with its retrospective nature and small sample size. Firstly, it was not possible to accurately determine the reasons that medical vs. surgical management was pursued in some patients. While patients that were more difficult to stabilize medically may have been taken for emergent surgery more frequently, it is likely that clinician experience/preference, staffing limitations and clinic protocols were major contributing factors in this decision-making process. Similarly, owner preference, expectations and financial limitations may also have contributed to the treatment choice in many cases. There were also nine patients in the “early surgical” group where the reason for early surgery could not be determined. Furthermore, the ultimate outcome of patients transferred to other clinics was not known, and is therefore a limitation of the study.

Due to the multi-center retrospective nature of the study, there were no standardized diagnostic and treatment protocols in place, and the quality of data collection was limited by what was documented in the clinical records. All patients were treated in a 24-h facility and components of medical management could have included intravenous fluid therapy, analgesia, oxygen therapy, transfusion therapy, antifibrinolytics and vasopressor support depending on the patient's individual needs. There is likely to have been significant variation in the knowledge and experience of the attending clinicians, as well as variable access to blood products, equipment and pharmacological agents between centers, and therefore directly comparable therapy may not have been instituted in all cases. For this reason, it is possible that some of the decompensated “early surgical” patients may have been able to be stabilized without surgery if more aggressive medical management was provided. This study did not investigate the timing of transfusions relative to hospitalization admission and surgical interventions, and did not investigate transfusion triggers or possible transfusions reactions. These factors could be considered for investigation in future studies of ASH. The frequency and quality of patient monitoring was also not standardized, but it is expected that unstable patients with ASH should receive frequent or constant monitoring during the initial treatment period. Further prospective studies are needed to assess individual treatment protocols and their effect on patient outcomes.

Abdominal fluid PCV equal to 10–25% of peripheral blood is considered consistent with hemoperitoneum (36). PCV measurement of the abdominal fluid was not routinely performed in all cases in this study, and therefore was not used as an inclusion criterion. Therefore, it is possible that patients with mixed fluid types may have been included in some instances. The presence of non-clotting blood in the abdomen, and consistent clinical pictures in all cases make this much less likely, however.

Additionally, there are no standardized criteria used to define cardiovascular stability in veterinary patients. The criteria used to evaluate cardiovascular stability in this study were extrapolated from normal ranges and recommendations listed in text books and review articles (18, 20), however these have not been independently validated. Blood pressure of 60 mm Hg (mean) or 90 mm Hg (systolic) were used to identify patients that remained hypotensive despite treatment, rather than used as a goal directed target for treatment. Lactate of 3.0 mmol/L was used as the cut off for stabilized patients, rather than a more conservative cut off of 2.5 because the upper limit of normal lactate range can vary from 2.5 to 3.0 mmol/L depending on the individual analyzer (37). Not all patients had both blood pressure and lactate recorded during hospitalization. It is possible that some patients would have been moved from the stable to unstable category if all three stabilization criteria were available for assessment of stability.

Unfortunately, the etiology of ASH was not able to be determined in cases that did not undergo surgical intervention. The volume of free abdominal fluid was also not able to be quantified from the clinical history in the majority of cases. This limits the utility of our findings as the likelihood of stabilization may be more dependent on etiology and degree of hemoperitoneum than on treatment group. Finally, the small sample size may have induced a type II error. A very small difference in stabilization was identified between groups in our cohort. If the true difference in proportion were indeed this small a sample size of over 20,000 cases would be required provide the power to detect such a difference. Further studies from different populations are recommended to determine if this result is consistent in other geographic locations and under different conditions.

## Conclusion

This preliminary study did not identify a significant difference in the ability to achieve cardiovascular stabilization in the first 12 h in dogs with ASH that underwent early surgical intervention vs. those receiving medical management only. Dogs over 20 kg, and dogs with a splenic source of hemorrhage were more likely to be stabilized. Patients in the “early surgical” group were more likely to receive a RBC transfusion that those in the “medical” group. At this time the decision on whether to pursue medical or surgical management should be made on a case-by-case basis. Further prospective, case-controlled studies or larger retrospective studies are indicated to determine whether medical or surgical management should be recommended in the initial treatment period of canine ASH.

## Data Availability Statement

The raw data supporting the conclusions of this article will be made available by the authors, without undue reservation.

## Ethics Statement

Ethical review and approval was not required for the animal study because this is a retrospective study involving data collection from case records. Handling of the animals involved in this study conforms to the Australian Code of Practice for the Care and Use of Animals for Scientific Purposes. Written informed consent for participation was not obtained from the owners because this is a retrospective study and did not include identifying patient details.

## Author Contributions

LW, YI, and MR contributed to conception and design of the study. LW, NF, RS, and AT collected data for the study. MR performed the statistical analysis. LW wrote the first draft of the manuscript. All authors contributed to manuscript revision, read, and approved the submitted version.

## Conflict of Interest

The authors declare that the research was conducted in the absence of any commercial or financial relationships that could be construed as a potential conflict of interest.

## Publisher's Note

All claims expressed in this article are solely those of the authors and do not necessarily represent those of their affiliated organizations, or those of the publisher, the editors and the reviewers. Any product that may be evaluated in this article, or claim that may be made by its manufacturer, is not guaranteed or endorsed by the publisher.
